# The healthy/unhealthy dietary pattern is associated with resting metabolic rate status among women with overweight/obesity

**DOI:** 10.1186/s12902-022-00958-z

**Published:** 2022-02-21

**Authors:** Sara Pooyan, Atieh Mirzababaei, Seyedeh Forough Sajjadi, Negin Badrooj, Yasaman Nasir, Somayeh Tajik, Masoumeh Fallahyekta, Mir Saeid Yekaninezhad, Khadijeh Mirzaei

**Affiliations:** 1 Department of Community Nutrition, School of Nutritional Sciences and Dietetics, Tehran University of Medical Sciences, P.O. Box 14155-6117, Tehran, Iran; 2 Department of Nutrition, Science and Research Branch, Islamic Azad University, Tehran, Iran; 3 Department of Epidemiology and Biostatistics, School of Public Health, Tehran University of Medical Sciences (TUMS), Tehran, Iran

**Keywords:** Resting metabolic rate, Obesity, Dietary pattern, Deviation of normal RMR

## Abstract

**Background:**

Although various dietary patterns have been indicated to be associated with the resting metabolic rate [RMR], limited data are available in this field. This study was therefore focused on the association between dietary patterns and resting metabolic rate among participants with overweight and obesity.

**Methods:**

This cross-sectional study was conducted on 304 women with overweight or obesity (BMI ≥ 25 kg/m^2^), aged 18–50. Anthropometric assessments, physical activity and biochemical measurements were assessed. RMR was also measured by means of indirect calorimetry. Dietary intake of participants was evaluated by 147-item semi-quantitative food frequency questionnaire [FFQ].

**Results:**

There was a significant association between higher adherence to the healthy dietary pattern [HDP] and RMR (*P* = 0.05), intakes of protein (*P* = 0.003), minerals (*P* = 0.001) as well as fat free mass [FFM] (*P* = 0.002), bone mineral content (*P* = 0.001), skeletal muscle mass (*P* = 0.001), soft lean mass (*P* = 0.002) and visceral fat area (*P* = 0.05). Also, there was a considerable association between higher adherence to the unhealthy dietary pattern [UHDP] and fasting blood sugar [FBS] (*P* = 0.05). Using multinomial logistic regression has been shown that the medium adherence to the HDP was marginally significant with decreased resting metabolic rate [Dec. RMR] group in crude model (OR: 0.54; 95% CI: 0.28–1.05, *P* = 0.07). After controlling for various confounders such as age, FFM, physical activity, and energy intake, the association between Dec. RMR group and the lowest quartile of the HDP (OR: 0.36; 95% CI: 0.14–0.91, *P* = 0.03) became significant as well as the association between Dec. RMR group and medium adherence to the HDP (OR: 0.42; 95% CI: 0.18–0.97, *P* = 0.04). The medium adherence to the UHDP in crude model was also significant with increased resting metabolic rate [Inc. RMR] group (OR: 2.59; 95% CI: 1.01–6.65, *P* = 0.04).

**Conclusions:**

Our study showed that there are significant associations between dietary patterns and RMR status.

**Supplementary Information:**

The online version contains supplementary material available at 10.1186/s12902-022-00958-z.

## Background

The prevalence of obesity, which has been shown to be relevant in the context of the development of various chronic diseases such as type 2 diabetes, cardiovascular diseases [CVDs] and various cancers, has been raised at an alarming rate [1, 2]; so that according to World Health Organization [WHO] estimates, in the recent decades, more than 650 million adults around the world, are obese [3, 4]. In 2002, about 70% of recorded mortalities in Iran have also shown to be associated with obesity-related chronic diseases [5, 6].

It is now well accepted that different transitions in dietary intakes as well as eating habits play prominent roles in increased rate of obesity, as a multi-factorial disorder [6–8]. Although prior studies have mostly investigated the intake of each nutrient in relation to various chronic diseases, it has been proved that combined effects of all of these individual nutrients are of higher importance. Therefore, the classification of the effects of all of these dietary components and their metabolic interactions is of both theoretical and practical significance. Regarding that, using dietary patterns allow a more comprehensive approach to prevent various health outcomes, such that recent investigations have shown that dietary patterns and obesity could be closely linked. Furthermore, some other studies have demonstrated that dietary patterns are among the major causes of obesity as well [9]. However, data in this regard were conflicting, so, more studies are needed to shed light on this issue.

The basal metabolic rate [BMR], as a complementary indicator of our body’s metabolism and compositions, has been shown to be the main component of every individual’s energy expenditure and could represent 60 to 75% of every participant’s energy intake. Hence, an expression of resting metabolism could be exactly related to higher risks of obesity and more importantly, could be used as a practical tool for the prevention of overweight and obesity [10–12]. Prior investigations have indicated that BMR as well as resting metabolic rate [RMR] were also directly associated with different dietary factors [13, 14]. For instance, a recent study was found that high-protein dietary pattern could significantly increase the BMR among patients with obesity [13], while another study could not represent these effects for poly unsaturated fatty acids [PUFA]-rich dietary patterns [14]. Moreover, other studies could not indicate any significant associations between different dietary patterns and BMR or RMR. Therefore, the effect of both BMR and RMR on the etiology of obesity is still under discussion [15].

Despite huge evidence in terms of the associations between dietary factors and RMR among western populations, we are aware of no Iranian study examining these relationships in this regard. Examining the roles of dietary patterns is particularly relevant for RMR since various mechanisms have been suggested that higher energy intake could be resulted in augmented body fat accumulation through reducing RMR. We hypothesized that evaluating the dietary pattern in relation to RMR is specifically more relevant for Iranian women since obesity is more prevalent among them which in return will clarify this issue more perfectly. Therefore, the purpose of the current study was to examine the association of dietary pattern with the deviation of normal RMR [DNR] among a group of Iranian adult women.

## Methods

### Study population

Three hundred and sixty women with body mass index [BMI] ≥ 25 were recruited from health centers of all the regions of West and Central Tehran, using community-based sampling based on-comfort sampling. We investigated obese individuals aged 18 to 50 who were generally in good health. Participants were excluded if they were taking medications or had history of type one diabetes mellitus. Smoking, alcohol consumption, pregnancy, lactation, and having vigorous exercise were considered as additional exclusion criteria. In addition, those participants who had been following an arbitrary special dietary pattern as well as those with chronic disease affecting their diet were excluded. Also, Women were excluded who did not respond to more than 70 food items in the food frequency questionnaire [FFQ], or who reported a total daily energy intake [EI] outside the range of 800–4200 kcal (3344–17,556 kJ). After an overnight (12 h) fast, volunteers attended to the clinical health centers in the Biochemical Laboratory of the School of Nutritional and Dietetics at Tehran University of Medical Sciences and standard anthropometric parameters were recorded. All participants provided signed informed written consent forms. The whole project was ethically approved by the Bioethics Committee of Tehran University of Medical Sciences, Tehran, Iran IR.TUMS.VCR.REC.1395.1593(95–04–161-33,833).

### Anthropometric assessment

We measured height, weight, as well as waist, and hip circumferences for each participant. All these measurements were performed by researchers who have extensive experience in compliance with WHO recommendations using a solar digital scale and a free-standing portable height meter. Participants were weighed without shoes and heavy outdoor clothing and weight recorded to the last 0.1 kg. Standing height was measured with a precision of 0.5 cm without shoes, by trained observers. BMI was also calculated by weight (kg)/ height (m) squared. Waist-to-hip ratio [WHR] was also calculated based on the dimensionless ratio of the circumference of the waist to that of the hips. WHR determines how much fat is stored on your waist, hips, and buttocks. We used BMI and WHR to classify overweight and obesity among participants. Based on WHO, a waist circumference of 102 cm (40 in.) or more in men, or 88 cm (35 in.) or more in women, is associated with obesity. Overweight and obesity were also defined as 25 ≤ BMI ≤ 29.9 kg/m^2^ and BMI ≥ 30 kg/m^2^, respectively.

### Measurement of resting metabolic rate and body composition

The body composition of all subjects was assessed with the use of Body Composition Analyzer *BC-418MA - Tanita* (United Kingdom) [16]. The device calculates BMI, weight, fat mass, body fat percentage, fat-free mass, visceral fat mass, muscle mass, abdominal fat mass; total body water foundation of data was obtained by dual energy X-ray absorptiometry [DXA] using Bioelectrical Impedance Analysis [BIA]. Subjects were barefoot when they were assessed by BIA device. We avoided taking measurements after severe exercise and waited until the individuals had rested enough.

RMR was measured by indirect calorimetry (spirometer METALYZERR 3B-R3, Cortex Biophysik GmbH, Leipzig, Germany). Based on the manufacturer’s instructions, prior to each test, gas exchange and ventilation was calibrated. The spiroergometric system with a high-resolution were conveyed by an amperometric solid electrolyte sensor for O2 assessment and an infrared sensor for CO2 evaluation, which recorded continuously through breath-by-breath gas analysis. A little division of breathing air was conducted through the volume flow sensor utilizing an ergonomically mask. The RMR was assessed by measuring the amount of O_2_ consumed and CO_2_ produced. The RMR was evaluated in the morning after a comfortable night’s sleep and following an overnight fasting. They asked participants to avoid severe exercise and alcohol or caffeine consumption for a day before RMR assessment. RMR was measured for 30 min in a quiet room and in steady position. The respiratory exchange ratio and oxygen uptake [VO2] were investigated within the average 20 min of the resting period predictive. RMR was determined using the Harris-Benedict equation, which considers age, weight and height for individuals.

In order to examine the association of body composition analysis, biochemical characters and RMR components with RMR status, the individuals were categorized to three groups: increased RMR [Inc. RMR], normal RMR and decreased RMR [Dec. RMR]. These groups were categorized based on the deviation of normal Resting Metabolic Rate [DNR]. In order to determine the DNR, we used indirect calorimetry (METALYZER 3B-R3). Moreover, we used fine standard deviation of variance for RMR to describe the DNR in this study. Then, we categorized the participants according to scoring of DNR. Such that, following definitions were considered for each of the mentioned groups: Inc. RMR (*n* = 37) (> 5% SD of normal RMR), normal RMR (*n* = 87) (− 5% SD < normal RMR < 5% SD), and Dec. RMR (*n* = 172) (normal RMR < − 5% SD).

### Dietary assessment

A semi quantitative food frequency questionnaire [FFQ] was used to assess the usual dietary intake of participants during the past year. The FFQ includes 147 items which are defined by a series of foods or beverages categorized into 9 major food groups. A standard serving size commonly consumed by Iranians and food frequency categories “daily/weekly/monthly” were used for all these items. The food items in the FFQ were categorized into 22 food groups. Then, we used the Nutritionist 4 software (First Data Bank, San Bruno, CA), which is based on United States Department of Agriculture [USDA] food composition table and modified for Iranian foods, to compute energy and nutrient content of foods. Validity and reliability of the FFQ have been confirmed previously [17].

### Assessment of other variables

The short-form International Physical Activity Questionnaire [IPAQ] was used to assess the physical activity. The validity and reliability of the IPAQ have been checked [18]. This questionnaire assesses particular types of activity over the past 7 days. Frequency and duration of physical activity were then converted to metabolic equivalent of tasks [METs]. We calculated blood pressure after 15-min rest in a chair-seated situation. Blood pressure was checked by the Automatic Inflate Blood Pressure Monitor (Samsung BA507S automatic digital blood pressure monitor, Samsung America, Inc).

### Measurement of biochemical parameters

Blood samples were accumulated between 8:00 and 10:00 in the morning, following 10 to 12 h of overnight fasting during the previous night. The serum after centrifuging and the liquated was stored at a temperature of − 80 °C. All measurements were taken at the biochemical laboratory of the school of nutritional science and dietetics. GOD/PAP [glucose oxidase phenol 4-Aminoantipyrine Peroxidase] method was used for the measured of fasting glucose and triglyceride levels as well as cholesterol levels were evaluated by the CHOD-PAP method. Moreover, total cholesterol [TC] levels and direct high-density lipoprotein [HDL] were measured by the Immunoinhibition assay. Aspartate aminotransferase [AST] and Serum alanine aminotransferase [ALT] were specified by athe International Federation of Clinical Chemistry and Laboratory Medicine method. Serum high-sensitivity C-reactive protein [hs-Crp] was determined using a high-sensitivity immunoturbidimetric assay (Hitachi 902 analyser; Hitachi Ltd., Tokyo, Japan). Pars Azmoon kit was used for all of the assessments (Pars Azmoon Inc. Tehran, Iran).

### Statistical analyses

In the current study, we first assessed the normal distribution of data using Kolmogorov-Smirnov test. Then, we obtained two major dietary patterns by the use of the factor analysis method (29). In this method, varimax rotation was constructed, according to the 22 food groups. Dietary patterns were identified based on the eigenvalues (> 1.5), KMO = 0.706, factor interpretability, scree plot, and variance explained. Then two dietary patterns were considered as Healthy and Unhealthy Dietary patterns. Then, we categorized participants into tertiles of adherence to the healthy and unhealthy dietary patterns divided into three groups (low, medium and high adherence to the healthy and unhealthy dietary patterns) and all statistical analyses were separately done for adherences to these dietary patterns. To describe our study population, we used mean ± standard deviation values. We applied one-way analysis of variance [ANOVA] to examine significant differences in continuous variables across these tertiles. To compare these differences again as well as adjusting the confounders’ effect at the same time, ANCOVA was applied. To determine the association of these adherences to dietary patterns with DNR, multinomial logistic regression was used in two models (crude and adjusted models). In the adjusted model, we adjusted for age (continuous) and energy intake as well as FFM and physical activity (METs/d). All confounding variables were established risk factors for DNR based on literature. The higher adherence to the both dietary patterns was considered as the reference category in all analyses. Results were demonstrated as odds ratios [ORs] and 95% confidence intervals [CIs] compared with the DNR groups. All statistical analyses were conducted using SPSS software (version 19.0; SPSS Inc., Chicago IL). *P* values were considered significant at < 0.05.

## Results

The mean age, weight, height, BMI, waist circumference and fat free mass [FFM] of the participants in this cross-sectional study were 36.49 years (SD = 8.38), 80.89 kg (SD = 12.45), 161.38 cm (SD = 5.90), 31.04 kg/m2 (SD = 4.31), 99.01 cm (SD = 10.05) and 46.80 kg (SD = 5.64), respectively (Table 1).

**Table 1:** Study population characteristics

	Minimum	Maximum	Mean ± SD
**Parameters**			
Age (years)	18	50	36.49 ± 8.38
Height (cm)	142	179	161.38 ± 5.90
Weight (kg)	59.50	136.60	80.89 ± 12.45
BMI	24.20	49.60	31.04 ± 4.31
**RMR parameters**			
RMR measure (kcal/day)	952.00	2480.00	1575.00 ± 259.71
RMR normal (kcal/day)	1425.00	2548.00	1720.40 ± 152.36
Deviation normal (%)	−44.00	40.00	−8.47 ± 12.59
V.O2 (L/min)	0.14	0.36	0.22 ± 0.037
V.CO2 (L/min)	0.01	0.30	0.19 ± 0.034
RQ	0.73	0.99	0.85 ± 0.412
RMR/kg (kcal/day/kg)	9.30	32.50	19.59 ± 3.09
RMR/BSA (kcal/day/m^2^)	552.00	1197.00	850.21 ± 113.89
**Body composition analysis**			
Protein (kg)	6.90	13.30	9.16 ± 1.10
Minerals (kg)	2.34	4.72	3.23 ± 0.41
Body fat mass (kg)	19.40	74.20	34.04 ± 8.69
Fat free mass (kg)	35.30	67.70	46.80 ± 5.64
Bone mineral content (kg)	1.82	3.93	2.67 ± 0.35
Skeletal muscle mass (kg)	18.90	37.90	25.69 ± 3.33
Soft lean mass (kg)	26.10	63.80	44.02 ± 5.37
Body fat percentage (%)	15.00	54.30	41.53 ± 5.48
Waist hip ratio	0.81	92.00	1.23 ± 5.24
Waist circumference (cm)	80.10	136.00	99.01 ± 10.05
Visceral fat area (cm^2^)	20.00	284.10	162.81 ± 39.32
Visceral fat level	7.00	208.40	16.64 ± 13.94
**Blood parameters**			
FBS (mg/dl)	67.00	137.00	87.49 ± 9.64
T-Chol (mg/dl)	104.00	344.00	185.30 ± 35.77
TG (mg/dl)	37.00	512.00	122.11 ± 69.29
HDL-C (mg/dl)	18.00	87.00	46.58 ± 10.86
LDL-C (mg/dl)	34.00	156.00	95.30 ± 24.12
GOT (UI/L)	6.00	60.00	18.05 ± 7.75
GPT (UI/L)	4.00	98.00	19.49 ± 13.83
hs-CRP (mg/L)	0.00	22.73	4.34 ± 4.62

*RMR* resting metabolic rate, *BMI* body mass index, *VO2* volume of oxygen, *VCO2* volume of carbon dioxide, *RQ* respiratory quotient, *FFM* fat-free mass, *FBS* fasting blood sugar, *TG* triglyceride, *T-chol* total cholesterol, *HDL-c* high-density lipoprotein cholesterol, *LDL-c* low-density lipoprotein cholesterol, *GOT* Aspartate Aminotransferase, *GPT* Alanine Aminotransferase, *hs-CRP* high-sensitivity C-reactive protein

*N* = 304

Data are indicated as Mean ± SD otherwise indicated

Factor analysis showed two main dietary patterns for our sample, food items and factor loadings for those are presented in Supplementary Table 1. The first factor identified as a healthy dietary pattern [HDP], was determined by high intakes of vegetables, fruits, nuts, low-fat dairy products, seasoning and condiments, red meat, vegetable starch, legumes, eggs, olive and olive oils and white meat, whereas Factor 2, the unhealthy dietary pattern [UHDP], was described by more consumption of high-fat dairy products, processed food, sweet and dessert, solid oil, whole grain, junk food and energetic drinks. Factor 1 explained 13.32% of the variance in intake while factor 2 explained 11.45% of the variance. Together, these two factors defined 24.77% of the variance.

Total participants were categorized based on the healthy and unhealthy pattern divided into three groups (Tables 2, 3). The differences between the low, medium and high adherence to the healthy and unhealthy pattern groups were analyzed by one-way ANOVA for body composition analysis, RMR components and biochemical characteristics. As shown in Table 2, the women with higher adherence to the healthy pattern had higher RMR (*P* = 0.05), protein (*P* = 0.003), minerals (*P* = 0.001), FFM (*P* = 0.002), bone mineral content (*P* = 0.001), skeletal muscle mass (*P* = 0.001), soft lean mass (*p* = 0.002), HDL-c (*P* = 0.83) and body fat mass (*P* = 0.32), and lower waist-hip ratio (*P* = 0.37), visceral fat area (*P* = 0.05), body fat percentage (*P* = 0.39), LDL (*P* = 0.58), hs- CRP (*P* = 0.67) and TC (*P* = 0.60) compared to the lower HDP group. But some findings were not statistically significant. There were no significant differences in terms of RMR/kg, RQ, visceral fat, body fat percentage and biochemical characteristics between the three groups (*P* > 0.05) (Table 2).

**Table 2:** Characteristics of the study participants between high, medium and low adherence of healthy dietary pattern

Adherence of HDP	Low	Medium	High	P^**2**^	P^**3,4**^
	***n*** = 132	***n*** = 159	***n*** = 159		
**Parameters**					
Age (years)	36.55 ± 8.85^**1**^	35.95 ± 8.20	37.04 ± 8.52	0.67	0.34
Height (cm)	160.15 ± 6.33 ^b, c^	161.19 ± 5.83 ^a, c^	162.53 ± 5.39 ^a, b^	**0.02**	0.68
Weight (kg)	79.10 ± 11.38 ^b, c^	79.92 ± 11.01 ^a, c^	83.13 ± 13.86 ^a, b^	**0.05**	0.39
BMI	30.98 ± 4.53	30.70 ± 3.70	31.47 ± 4.69	0.46	0.95
**RMR parameters**					
RMR measure (kcal/day)	1558.4 ± 253.05	1570.8 ± 244.28	1602.4 ± 280.93	0.48	0.43
RMR normal (kcal/day)	1700.8 ± 142.41 ^c^	1706.1 ± 126.20	1749.4 ± 175.6 ^a^	**0.05**	0.27
Deviation normal (%)	−8.42 ± 12.98	−7.91 ± 12.23	−8.43 ± 12.96	0.95	0.48
V.O2 (L/min)	0.22 ± 0.03	0.22 ± 0.03	0.22 ± 0.04	0.43	0.58
V.CO2 (L/min)	0.19 ± 0.03	0.19 ± 0.03	0.19 ± 0.03	0.83	0.22
RQ	0.85 ± 0.03	0.85 ± 0.04	0.84 ± 0.04	0.23	0.62
RMR/kg (kcal/day/kg)	19.78 ± 3.40	19.85 ± 2.81	19.32 ± 3.11	0.44	0.53
RMR/BSA (kcal/day/m^2^)	850.76 ± 119.50	855.39 ± 108.58	850.99 ± 116.30	0.95	0.47
**Body composition**					
Protein (kg)	8.90 ± 0.99 ^b, c^	9.14 ± 1.09 ^a, c^	9.44 ± 1.11 ^a, b^	**0.003**	0.69
Minerals (kg)	3.11 ± 0.38 ^c^	3.21 ± 0.40 ^a, c^	3.34 ± 0.41 ^a, b^	**0.001**	0.25
Body fat mass (kg)	33.78 ± 8.58	33.21 ± 7.44	35.03 ± 9.82	0.32	0.81
Fat free mass (kg)	45.42 ± 5.06 ^b, c^	46.65 ± 5.51 ^a, c^	48.25 ± 5.81 ^a, b^	**0.002**	**0.02**
Bone mineral content (kg)	2.58 ± 0.33 ^b, c^	2.65 ± 0.33 ^a, c^	2.76 ± 0.35 ^a, b^	**0.001**	0.16
Skeletal muscle mass (kg)	24.87 ± 2.98 ^b, c^	25.60 ± 3.28 ^a, c^	26.57 ± 3.42 ^a, b^	**0.001**	0.35
Soft lean mass (kg)	42.83 ± 4.77 ^b, c^	43.68 ± 5.37 ^a^	45.48 ± 5.48 ^a, b^	**0.002**	0.37
Body fat percentage (%)	42.15 ± 5.55	41.25 ± 4.93	41.15 ± 6.07	0.39	0.27
Waist hip ratio	1.87 ± 9.29	0.93 ± 0.04	0.93 ± 0.05	0.37	0.52
Waist circumference (cm)	97.92 ± 9.40	98.51 ± 9.24 ^a, c^	100.4 ± 11.27	0.18	0.44
Visceral fat area (cm^2^)	178.04 ± 173.51	160.90 ± 36.07	166.01 ± 42.83	0.50	0.49
Visceral fat level	17.53 ± 20.04	15.41 ± 3.26	17.06 ± 13.99	0.55	0.51
**Blood parameters**					
FBS (mg/dl)	89.18 ± 11.33	86.51 ± 9.23 ^a^	86.84 ± 8.00	0.15	0.12
T-Chol (mg/dl)	187.26 ± 34.76	181.89 ± 40.71	186.32 ± 33.09	0.60	0.82
TG (mg/dl)	114.64 ± 55.17	122.45 ± 78.34	123.11 ± 75.11	0.40	0.28
HDL-C (mg/dl)	46.88 ± 10.00	46.28 ± 10.66	47.26 ± 11.86	0.83	0.32
LDL-C (mg/dl)	99.00 ± 24.46	92.75 ± 24.23	96.33 ± 24.01	0.58	0.41
GOT (UI/L)	17.02 ± 5.27	17.67 ± 6.47	18.73 ± 9.54	0.32	0.40
GPT (UI/L)	18.18 ± 9.39	18.84 ± 11.47	20.38 ± 16.80	0.54	0.90
hs-CRP (mg/L)	4.65 ± 4.96	4.34 ± 4.78	3.99 ± 4.24	0.67	0.24

*HDP* Healthy dietary pattern, *RMR* resting metabolic rate, *BMI* body mass index, *VO2* volume of oxygen, *VCO2* volume of carbon dioxide, *RQ* respiratory quotient, *FFM* fat-free mass, *FBS* fasting blood sugar, *TG* triglyceride, *T-chol* total cholesterol, *HDL-c* high-density lipoprotein cholesterol, *LDL-c* low-density lipoprotein cholesterol, *GOT* Aspartate Aminotransferase, *GPT* Alanine Aminotransferase, *hs-CRP* high-sensitivity C-reactive protein; *N* = 304

^1^ Mean ± SD

^2^ *P*-values are resulted from ANOVA (Analysis of variance)

^3^ *P*-values are resulted from ANCOVA

^4^After adjustment for age, FFM, energy intake, and physical activity (METs/d)

^a^ Significant compared with low adherence

^b^ Significant compared with medium adherence

^c^ Significant compared with high adherence

**Table 3:** Characteristics of the study participants between low, medium and high adherence of unhealthy dietary pattern

Adherence of UHDP	Low	Medium	High	P^**2**^	P^**3,4**^
	***n*** = 132	***n*** = 159	***n*** = 159		
**Parameters**					
Age (years)	37.85 ± 7.57 ^b, c^	37.01 ± 9.13 ^a, c^	34.69 ± 8.53 ^a, b^	**0.02**	0.16
Height (cm)	161.03 ± 6.52	160.61 ± 5.58	162.24 ± 5.56	0.13	0.56
Weight (kg)	80.58 ± 11.00	79.65 ± 12.32	81.91 ± 13.27	0.43	0.55
BMI	31.08 ± 3.93	30.78 ± 4.20	31.29 ± 4.82	0.71	0.60
**RMR parameters**					
RMR measure (kcal/day)	1544.8 ± 255.81	1580.1 ± 276.87	1606.1 ± 243.24	0.26	0.42
RMR normal (kcal/day)	1709.1 ± 132.13 ^b, c^	1696.0 ± 143.50 ^a, c^	1750.8 ± 170.09 ^a, b^	**0.03**	0.23
Deviation normal (%)	−9.72 ± 11.90	−6.85 ± 14.05	−8.20 ± 11.93	0.29	0.29
V.O2 (L/min)	0.22 ± 0.03	0.22 ± 0.03	0.22 ± 0.03	0.38	0.53
V.CO2 (L/min)	0.18 ± 0.03	0.19 ± 0.03	0.19 ± 0.03	0.20	0.39
RQ	0.85 ± 0.04	0.85 ± 0.04	0.85 ± 0.03	0.86	0.73
RMR/kg (kcal/day/kg)	19.21 ± 2.76	20.13 ± 3.40	19.61 ± 3.11	0.12	0.14
RMR/BSA (kcal/day/m^2^)	835.04 ± 110.75	863.53 ± 127.53	858.51 ± 102.79	0.18	0.29
**Body composition**					
Protein (kg)	9.16 ± 1.16	9.10 ± 1.07	9.22 ± 1.02	0.75	0.51
Minerals (kg)	3.22 ± 0.45	3.19 ± 0.40	3.25 ± 0.37	054	0.65
Body fat mass (kg)	33.66 ± 7.39	33.10 ± 8.64	35.25 ± 9.75	0.20	0.17
Fat free mass (kg)	46.81 ± 5.95	46.41 ± 5.50	47.12 ± 5.30	0.67	0.29
Bone mineral content (kg)	2.67 ± 0.38	2.63 ± 0.33	2.69 ± 0.31	0.47	0.66
Skeletal muscle mass (kg)	25.69 ± 3.53	25.52 ± 3.26	25.82 ± 3.11	0.81	0.17
Soft lean mass (kg)	44.14 ± 5.58	43.45 ± 5.33	44.41 ± 5.02	0.42	0.33
Body fat percentage (%)	41.63 ± 4.97	41.03 ± 5.51	41.88 ± 6.08	0.55	0.26
Waist hip ratio	0.93 ± 0.05	1.86 ± 9.24	0.93 ± 0.05	0.37	0.50
Waist circumference (cm)	98.89 ± 9.05	98.08 ± 10.34	99.91 ± 10.67	0.44	0.30
Visceral fat area (cm^2^)	163.08 ± 34.47	175.56 ± 172.92	166.16 ± 43.84	0.69	0.64
Visceral fat level	15.64 ± 3.10	16.52 ± 14.02	17.81 ± 19.94	0.56	0.52
**Blood parameters**					
FBS (mg/dl)	85.87 ± 8.11^b, c^	89.44 ± 11.52 ^a, c^	87.37 ± 8.84 ^a, b^	**0.05**	**0**.**01**
T-Chol (mg/dl)	185.04 ± 35.53	185.85 ± 39.03	184.56 ± 34.54	0.97	0.47
TG (mg/dl)	122.84 ± 76.18	129.68 ± 74.78	114.73 ± 58.00	0.41	0.38
HDL-C (mg/dl)	46.75 ± 10.84	46.36 ± 11.65	47.32 ± 10.10	0.85	0.65
LDL-C (mg/dl)	95.34 ± 23.83	94.81 ± 24.85	94.89 ± 24.22	0.98	0.81
GOT (UI/L)	17.86 ± 8.01	17.77 ± 6.07	17.83 ± 7.84	0.99	0.83
GPT (UI/L)	18.53 ± 13.72	19.32 ± 10.98	19.67 ± 14.06	0.84	0.93
hs-CRP (mg/L)	3.92 ± 4.42	4.45 ± 5.02	4.63 ± 4.55	0.59	0.26

*UHDP* unhealthy dietary pattern, *RMR* resting metabolic rate, *BMI* body mass index, *VO2* volume of oxygen, *VCO2* volume of carbon dioxide, *RQ* respiratory quotient, *FFM* fat-free mass, *FBS* fasting blood sugar, *TG* triglyceride, *T-chol* total cholesterol, *HDL-c* high-density lipoprotein cholesterol, *LDL-c* low-density lipoprotein cholesterol, *GOT* Aspartate Aminotransferase, *GPT* Alanine Aminotransferase, *hs-CRP* high-sensitivity C-reactive protein. *N* = 304

^1^ Mean ± SD

^2^ *P*-values are resulted from ANOVA (Analysis of variance)

^3^ *P*-values are resulted from ANCOVA

^4^After adjustment for age, FFM, energy intake, and physical activity (METs/d)

^a^ Significant compared with low adherence

^b^ Significant compared with medium adherence

^c^ Significant compared with high adherence

The individuals with higher adherence to the UHDP had higher waist circumference (*p* = 0.44), Visceral fat (*P* = 0.69), FBS (*P* = 0.05), GPT (*P* = 0.84) and hs-CRP (*P* = 0.59) compare to low unhealthy pattern group. However, there were no significant association in these items (*P* > 0.05). After adjustment for the confounders including age, FFM, physical activity and energy intake was observed the significant difference in FBS (*P* = 0.01) (Table 3).

Individuals were categorized into three groups; Inc. RMR, normal RMR and Dec. RMR according to DNR scores. The differences between the normal, Dec. and Inc. RMR groups were analyzed by one-way ANOVA tests. Individuals in Inc. RMR group had significantly higher height (*P* < 0.006), RMR (*P* < 0.0001), RMR/kg (*p* < 0.0001), VO_2_ (*P* < 0.0001), RMR/BSA (*P* < 0.0001) as well as higher dietary intake of minerals (*P* < 0.0001), protein (*P* < 0.0001) and higher bone mineral content (*P* < 0.0001), soft lean mass (*P* < 0.0001), skeletal muscle mass (*P* < 0.0001), body fat percentage (*P* = 0.001) compared to Dec. RMR group. All significant outcomes remained robust after adjusting for the confounders including age, energy intake, physical activity, and FFM. Furthermore, body fat mass (*P* = 0.01) has become also significant.

The association between HDP and UHDP and RMR across DNR are shown in Table 4. We considered the higher adherence to the dietary pattern as reference category. The possibility Dec. RMR in the women with medium adherence to the HDP was higher, compared to women with higher adherence to the HDP. The medium adherence to the HDP was marginally significant with Dec. RMR group in crude model (OR: 0.54; 95% CI: 0.28–1.05, *P* = 0.07). After controlling for the confounders like age, FFM, physical activity, and energy intake, the association between Dec. RMR group and the lowest quartile of the HDP (OR: 0.36; 95% CI: 0.14–0.91, *P* = 0.03) became significant and also, the association between Dec. RMR group and medium adherence to the HDP (OR: 0.42; 95% CI: 0.18–0.97, *P* = 0.04).

**Table 4:** The association between dietary pattern and RMR across deviation of normal RMR

		DNR	β	OR (95% CI)	P^¶^
**Healthy diet**					
**Low**	Crude model	Dec. RMR	− 0.42	0.65 (0.33–1.27)	0.21
		Inc. RMR	−0.43	0.65 (0.25–1.66)	0.36
	Adjusted model ^a^	Dec. RMR	−1.00	0.36 (0.14–0.91)	**0.03**
		Inc. RMR	−0.64	0.52 (0.14–1.92)	0.32
**Medium**	Crude model	Dec. RMR	−0.61	0.54 (0.28–1.05)	**0.07**
		Inc. RMR	−0.81	0.44 (0.16–1.17)	0.10
	Adjusted model	Dec. RMR	−0.84	0.42 (0.18–0.97)	**0.04**
		Inc. RMR	−0.68	0.50 (0.15–1.66)	0.26
**Unhealthy diet**					
**Low**	Crude model	Dec. RMR	0.15	1.16 (0.62–2.19)	0.62
		Inc. RMR	−0.18	0.83 (0.27–2.53)	0.74
	Adjusted model	Dec. RMR	0.0001	0.1 (0.42–2.35)	0.99
		Inc. RMR	−0.86	0.42 (0.09–1.88)	0.25
**Medium**	Crude model	Dec. RMR	−0.03	0.97 (0.50–1.85)	0.92
		Inc. RMR	−0.95	2.59 (1.01–6.65)	**0.04**
	Adjusted model	Dec. RMR	−0.10	0.89 (0.39–2.02)	0.79
		Inc. RMR	0.87	2.39 (0.73–7.80)	0.14

*RMR* resting metabolic rate, *DNR* deviation of normal resting metabolic rate, *Dec. RMR* decreased of normal status of resting metabolic rate, *Inc. RMR* increased of normal status of resting metabolic rate, *OR* odd ratio, *CI* confidence interval.

*N* = 304

¶ P-values are resulted from multinomial logistic regression

The higher adherence to the both dietary patterns was considered as the reference category in all analyses

Results were demonstrated as odds ratios [ORs] and 95% confidence intervals [CIs] compared with the DNR groups. ^a^ adjusted model: adjusted for age, FFM, physical activity (METs/d), energy intake

The medium adherence to the UHDP in crude model was significant with Inc. RMR group (Inc. RMR: OR: 2.59; 95% CI: 1.01–6.65, *P* = 0.04). However, after controlling for confounders, no significant association was remained with Dec. RMR (OR: 0.89; 95% CI: 0.39–2.02, *P* = 0.79) and Inc. RMR (OR: 2.39; 95% CI:0.73–7.80, *P* = 0.14).

## Discussion

Several studies suggested that overweight and obesity have been rapidly increased worldwide. This cross-sectional study, in order to deal with the complications of obesity and overweight, tried to find the mediatory role of HDP and UHDP on RMR which is one of the major factors for controlling body weight. Besides, HDP and UHDP might be advantageous to decrease the development of overweight and obesity. Therefore, we investigated the effects of HDP and UHDP on the possible link between DNR and obesity.

This cross-sectional study has shown that dietary patterns can be associated with DNR among women with overweight and obesity. Two dietary patterns were identified, by using the factor analysis technique; these were HDP and UHDP. It was found that higher adherence to the HDP could be associated with several factors, including FFM, protein, minerals, bone mineral content, skeletal muscle mass and soft lean mass. This result may have been attributed to the higher intake of protein, soluble and insoluble fiber, omega-3 fatty acid, calcium, magnesium, iron, potassium, and vitamin C in healthy dietary pattern group [19–22]. This finding was contradictory with previous observations that Mediterranean pattern was not related to FFM, muscle mass index and total hip BMD [19, 21]. Also, it was shown that higher adherence to the UHDP could be linked with more FBS which might be due to increased intake of carbohydrate, sweets and dessert. Our findings were in line with previous studies that an unhealthy dietary pattern was associated with increased fasting plasma glucose [22].

The other outcomes are that enhanced RMR is strongly related to higher height, VO_2,_ RMR, RMR/kg, RMR/BSA, protein, minerals, skeletal muscle mass, bone mineral content, soft lean mass, body fat percentage. These results were consistent with the former study that demonstrated fat mass was related to an increased metabolic rate among women with up to 40% body fat. Increased fat mass has a considerable influence on the metabolic function. Fat mass could influence the metabolic rate by chronic changes in hormonal concentrations. Skeletal muscle is the easily manipulated contributor to RMR and also, by altered substrate oxidation and metabolic rate. Muscle mass is the main location for substrate oxidation and is associated with improved glucose regulation and insulin, but the association between body composition and the metabolic function is still unknown. The previous study of RMR and visceral fat presented that visceral fat distribution plays an important role in determining the RMR. But in another study, this correlation was not observed.

We found a significant association HDP and UHDP with DNR’s groups in the present study. The HDP, including the frequent intake of vegetables, fruits, nuts, low-fat dairy products, eggs, red and white meat, olive and legumes. We observed that after adjustment for age, physical activity, FFM, and energy intake; risk of RMR decreasing among women with the lower and medium adherence to the HDP was higher, compared to higher adherence to the HDP. Moreover, odds of RMR increasing among women with the medium adherence to the UHDP was higher, compared to greater adherence to the UHDP.

The present study has several strengths. As far as we know, this is the first study which determine the possible relationship of HDP and UHDP with DNR among Iranian women with overweight and obesity. However, during the interpretation of our findings, some limitations should be also noticed. The main limitation is the cross-sectional design of our study, which prohibit us inferring causality. Therefore, further prospective studies are needed to confirm our findings. As the other limitation of this study, the little number of participants in the same-sex sample could be mentioned. In addition, measurement error is another potential limitation, as is in all dietary assessment methods. Due to using FFQ to assess usual dietary intakes, misclassification of study individuals is another concern. However, we used a validated FFQ for assessment of dietary intakes. Furthermore, we cannot exclude residual confounders despite adjusting for a wide range of potential confounders.

## Conclusions

In conclusion, our study has shown significant associations between dietary patterns and RMR status. Our findings should be confirmed by future prospective studies.

## Supplementary Information

**Additional file 1: Supplementary Table 1.** Food groups used in the factor analysis and factor loadings for each of the identified dietary patterns.

## Data Availability

The data that support the findings of this study are available from Khadijeh Mirzaei but restrictions apply to the availability of these data, which were used under license for the current study, and so are not publicly available. Data are, however, available from the authors upon reasonable request and with permission of Khadijeh Mirzaei.

## Abbreviations

RMR: Resting Metabolic Rate
FFQ: Food Frequency Questionnaire
HDP: Healthy Dietary Pattern
FFM: Fat Free Mass
UHDP: Unhealthy Dietary Pattern
FBS: Fasting Blood Sugar
Dec. RMR: Decreased Resting Metabolic Rate
Inc. RMR: Increased Resting Metabolic Rate
CVDS: Cardiovascular Diseases
WHO: World Health Organization
BMR: Basal Metabolic Rate
PUFA: Poly Unsaturated Fatty Acids
BMI: Body Mass Index
EI: Energy Intake
WHR: Waist-To-Hip Ratio
DXA: Dual Energy X-Ray Absorptiometry
BIA: Bioelectrical Impedance Analysis
DNR: Deviation Of Normal Resting Metabolic Rate
USDA: United States Department Of Agriculture
IPAQ: International Physical Activity Questionnaire
Mets: Metabolic Equivalent Of Tasks
GOD/PAP: Glucose Oxidase Phenol 4-Aminoantipyrine Peroxidase
TC: Total Cholesterol
HDL: High-Density Lipoprotein
AST: Aspartate Aminotransferase
ALT: Serum Alanine Aminotransferase
Hs-Crp: High-Sensitivity C-Reactive Protein
ANOVA: One-Way Analysis Of Variance
