# Supplementary material for: The healthy/unhealthy dietary pattern is associated with resting metabolic rate status among women with overweight/obesity
Source: BMC Endocr Disord. 2022 Feb 21;22:45. doi: 10.1186/s12902-022-00958-z (PMC8862357; doi:10.1186/s12902-022-00958-z)
Supplement: Supplementary file 1 — Additional file 1: Supplementary Table 1. Food groups used in the factor analysis and factor loadings for each of the identified dietary patterns. [file 12902_2022_958_MOESM1_ESM.docx]

**Supplementary Table 1. Food groups used in the factor analysis and factor loadings for each of the identified dietary patterns.**

| Food groups | Food items | Healthy | Unhealthy |
| --- | --- | --- | --- |
| Whole grain White breads (Lavash, baguette), cooked rice, reshteh, vermishel,  Dark breads, (Barbari, Sangak, Taftoon), cooked barley or bulgur,  spaghetti - 0.50 | | | |
| Legumes Lentil, bean, chickpea, cooked broad bean, mung bean, split pea 0.39 - | | | |
| Vegetables Leafy vegetables (raw and cooked), lettuce, celery, cucumber, spinach, 0.72 -  bell pepper, mushroom, tomato, zucchini, eggplant, pumpkin,  carrot (raw and cooked), garlic, onion (raw and fried), turnip,  green chilies, cruciferous vegetables | | | |
| Fruits Watermelon, honeydew melon and cantaloupe, persian melon, pear, 0.58 -  apricot, apple, cherry and sour cherry, peach, nectarine, Greengages,  fig, grapes, kiwi, grapefruit, orange, persimmon, tangerine, banana,  pomegranate, dates, plums, strawberry, sweet lemon, lime lemon  mulberry, dried fig, dried mulberry, dried fruits, raisins, fruit juices  (orange, apple, and honeydew) | | | |
| Nuts Peanut, almond, walnut, pistachio, hazelnut, seeds, soya 0.50 - | | | |
| Low-fat dairy Low-fat milk, doogh (yogurt drink), plain yogurt, kashk, Cheese 0.48 -  Products | | | |
| High-fat dairy High-fat milk, chocolate milk, cheese, cream cheese, cream, - 0.53  products traditional ice-cream, non-traditional ice-cream, high-fat yogurt,  concentrated yogurt | | | |
| Condiments ketchup, lime juice, Salt, Salted pickles, Pickled cucumber, 0.48 -  other pickles | | | |
| Seasoning Spices 0.406 - | | | |
| Red meat Red meats (beef or lamb), mincemeat 0.48 - | | | |
| White meat poultry, fishes, canned tuna fish 0.36 - | | | |
| Vegetable starch corn, green pea, green bean, cooked potato 0.45 - | | | |
| Eggs Eggs 0.37 - | | | |
| Olive Olives, olive oil 0.36 - | | | |
| Processed food Hamburger, sausage, Pizza, French fries - 0.51 | | | |
| Sweet and dessert Cakes, chocolates, halvah, Sweets, gaz, sohan, candy, - 0.56  Biscuit, Honey, Jams, Sugar, Compote the fruit | | | |
| Solid oil solid oils, animal fats, butter, margarine - 0.39 | | | |
| Liquid oil Mayonnaise, Liquid oil - - | | | |
| Junk food crackers, potato chips, cheese puffs - 0.56 | | | |
| Sweet beverage Soft drinks, commercial fruit juice 0.69 | | | |
| Tea and coffee Tea and coffee - - | | | |
| Organ meats Brain, tongue, feet, head, liver kidney and heart, offal  and rennet - - | | | |
| Total variance explained (%) 13.32 11.45 | | | |
